# IGF‐1 deficiency impairs neurovascular coupling in mice: implications for cerebromicrovascular aging

**DOI:** 10.1111/acel.12372

**Published:** 2015-07-14

**Authors:** Peter Toth, Stefano Tarantini, Nicole M. Ashpole, Zsuzsanna Tucsek, Ginger L. Milne, Noa M. Valcarcel‐Ares, Akos Menyhart, Eszter Farkas, William E. Sonntag, Anna Csiszar, Zoltan Ungvari

**Affiliations:** ^1^ Donald W. Reynolds Department of Geriatric MedicineReynolds Oklahoma Center on AgingUniversity of Oklahoma Health Sciences CenterOklahoma CityOK73104USA; ^2^ Department of Neurosurgery and Szentagothai Research CenterMedical SchoolUniversity of PecsPecs7624Hungary; ^3^ Department of PhysiologyUniversity of Oklahoma Health Sciences Center940 S.L. Young Blvd. Rm. 653 Oklahoma City73104OKUSA; ^4^ Division of Clinical PharmacologyVanderbilt University Medical Center D‐3100Medical Center NorthNashvilleTNUSA; ^5^ Department of Medical Physics and InformaticsFaculty of Medicine and Faculty of Science and InformaticsUniversity of SzegedSzeged6720Hungary; ^6^ The Peggy and Charles Stephenson Cancer CenterUniversity of Oklahoma Health Sciences CenterOklahoma CityOK73104USA; ^7^ Department of Pulmonology1125 Budapest, Diós árok 1/c Semmelweis UniversityBudapestHungary

**Keywords:** arachidonic acid metabolites, astrocyte, endothelial dysfunction, functional hyperemia, Insulin‐like growth factor‐1, neurovascular uncoupling, nitric oxide, somatomedin C, vascular aging, vascular cognitive impairment

## Abstract

Aging is associated with marked deficiency in circulating IGF‐1, which has been shown to contribute to age‐related cognitive decline. Impairment of moment‐to‐moment adjustment of cerebral blood flow (CBF) via neurovascular coupling is thought to play a critical role in the genesis of age‐related cognitive impairment. To establish the link between IGF‐1 deficiency and cerebromicrovascular impairment, neurovascular coupling mechanisms were studied in a novel mouse model of IGF‐1 deficiency (*Igf1* ^*f/f*^‐TBG‐Cre‐AAV8) and accelerated vascular aging. We found that IGF‐1‐deficient mice exhibit neurovascular uncoupling and show a deficit in hippocampal‐dependent spatial memory test, mimicking the aging phenotype. IGF‐1 deficiency significantly impaired cerebromicrovascular endothelial function decreasing NO mediation of neurovascular coupling. IGF‐1 deficiency also impaired glutamate‐mediated CBF responses, likely due to dysregulation of astrocytic expression of metabotropic glutamate receptors and impairing mediation of CBF responses by eicosanoid gliotransmitters. Collectively, we demonstrate that IGF‐1 deficiency promotes cerebromicrovascular dysfunction and neurovascular uncoupling mimicking the aging phenotype, which are likely to contribute to cognitive impairment.

## Introduction

Vascular cognitive impairment (VCI) in the aging population poses a serious challenge to developed countries around the world. With the expansion of the aging population, understanding potentially reversible and preventable vascular contributions to age‐related cognitive impairment and dementia is of critical importance.

There is increasing evidence that in addition to pathologies affecting the larger cerebral arteries (e.g. atherosclerosis), pathophysiological alterations of the cerebral microcirculation have a critical role in age‐related decline in brain function (Gorelick *et al*., 2011). Among them, age‐related functional changes in the neurovascular unit that have the potential to impair local regulation of cerebral blood flow are of great importance (Iadecola *et al*., 2009). The energy requirements of neurons are high. Yet, the brain contains little energy reserves and, during neuronal activation, there is a need for rapid increases in oxygen and glucose delivery. This is ensured by neurovascular coupling, a vital feed‐forward control mechanism involving neuronal signaling via neurotransmitters, which adjusts local cerebral blood flow (CBF) to the energy requirements of activated neurons. The resulting functional hyperemia is responsible for maintenance of an optimal local microenvironment in the cerebral tissue by increasing delivery of oxygen and glucose and removal of potentially deleterious by‐products of cerebral metabolism. Aging is associated with significant impairment of functional hyperemia (termed ‘neurovascular uncoupling’), and the ensuing disruption of the cerebral microenvironment likely contributes to impairment of higher cerebral function in elderly patients and aged laboratory animals (Zaletel *et al*., 2005; Park *et al*., 2007; Topcuoglu *et al*., 2009; Fabiani *et al*., 2013; Sorond *et al*., 2013; Stefanova *et al*., 2013; Toth *et al*., 2014a). Yet, the specific age‐related mechanisms responsible for neurovascular uncoupling are not yet understood.

There is increasing evidence suggesting that neuroendocrine mechanisms have an important role in age‐related vascular alterations (Ungvari & Csiszar, 2012; Sonntag *et al*., 2013). In particular, the age‐related decline in circulating insulin‐like growth factor‐1 (IGF‐1) levels appears to contribute significantly to age‐related microvascular changes and cognitive decline (reviewed recently in Sonntag *et al*. (2013)). Neurovascular coupling depends on an intact functional network of neurons, vascular endothelial cells, and astrocytes (Attwell *et al*., 2010; Chen *et al*., 2014). Although these cell types abundantly express IGF‐1 receptors and are known targets of IGF‐1 (Sonntag *et al*., 2013), the role of IGF‐1 in the regulation of functional hyperemia in response to neuronal activation is not well understood. The cellular mechanisms underlying neurovascular coupling include endothelial production of nitric oxide (NO) (Ma *et al*., 1996; Stobart *et al*., 2013; Chen *et al*., 2014) as well as astrocytic production of vasodilator metabolites of arachidonic acid, including epoxygenase‐derived epoxyeicosatrienoic acids (EETs) and cyclooxygenase‐derived prostaglandins (Peng *et al*., 2002; Zonta *et al*., 2003; Takano *et al*., 2006). Importantly, previous studies demonstrate that IGF‐1 deficiency leads to endothelial dysfunction and impaired bioavailability of NO in the peripheral circulation (reviewed in Ungvari & Csiszar (2012)). IGF‐1 was also shown to regulate cellular arachidonic acid metabolism (Tahara *et al*., 1991; Berenbaum *et al*., 1994; Damke *et al*., 1994; Sharma *et al*., 1997). Despite these advances, the effects of IGF‐1 deficiency on the cerebral microcirculation and mediation of neurovascular coupling by NO, EETs, and eicosanoid gliotransmitters remain elusive.

This study was designed to test the hypotheses that IGF‐1 regulates synthesis/release of NO and vasodilator eicosanoid gliotransmitters in the cerebral microcirculation and that low circulating IGF‐1 levels impair neurovascular coupling in the brain, mimicking the aging phenotype. To test our hypotheses, we used a novel mouse model of adult‐onset, isolated endocrine IGF‐1 deficiency induced by adeno‐associated viral knockdown of IGF‐1 specifically in the liver of postpubertal mice using Cre‐lox technology (*Igf1* ^*f/f*^ + TBG‐Cre‐AAV8) (Toth *et al*., 2014b). Neurovascular coupling, synthesis of eicosanoid gliotransmitters, astrocytic gene expression, and cerebromicrovascular endothelial function were tested. To substantiate our findings, behavioral studies known to be sensitive for neurovascular uncoupling (tests indicative for learning and memory) were conducted.

## Results

### IGF‐1 deficiency impairs neurovascular coupling and cognitive function

**Figure 1:**
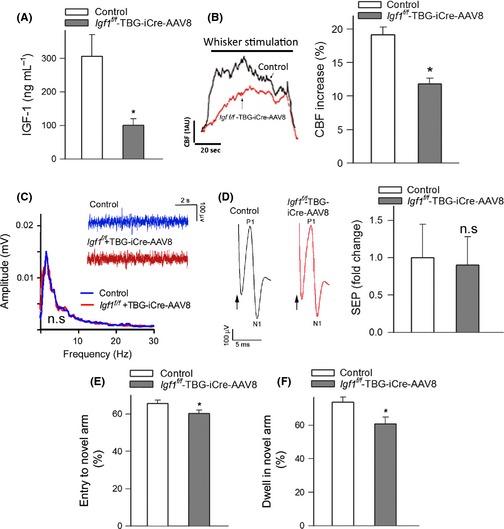
IGF‐1 deficiency impairs neurovascular coupling and cognitive function. Panel A shows that adeno‐associated viral knockdown of hepatic *Igf1 (Igf1* ^*f/f*^ + TBG‐Cre‐AAV8) decreases significantly the level of circulating IGF‐1 compared to control animals (*Igf1* ^*f/f*^ + TBG‐eGFP‐AAV8) (*P < 0.05 vs. control). (B) Representative traces of cerebral blood flow (CBF) measured with a laser Doppler probe above the whisker barrel cortex during contralateral whisker stimulation (5 Hz) in control and IGF‐1‐deficient mice. 1 AU corresponds to ~5% increase in CBF from baseline. Right panel depicts the summary data of the CBF responses (ΔAUC as % of baseline; *n *= 12, *P < 0.001 vs. control). (C–D) Spontaneous and evoked neural activity is not altered in IGF‐1‐deficient mice. (C) The amplitude and frequency distribution of neocortical electrical activity are nearly identical in control and IGF‐1‐deficient mice (inlet shows original recording of electrocorticograms, *n *= 6, *P* = 0.4). (D) The somatosensory evoked potential (SEP) responses in the somatosensory cortex evoked by contralateral whisker pad stimulation are comparable in control and IGF‐1‐deficient mice. The arrow indicates the application of the stimulus. The amplitude of the negative wave of the field potentials (N1) does not differ between control and IGF‐1‐deficient mice (*n *= 6, *P* = 0.6). (E–F) Spatial memory testing of mice in Y‐maze. The IGF‐1‐deficient animals *(Igf1* ^*f/f*^ + TBG‐Cre‐AAV8) exhibited impaired spatial memory as shown by the decreased number of entries in novel arm (E; * *P* = 0.001 vs. control) and shorter exploratory time spent in novel arm of the Y‐maze during retrieval trial (F; and *P* = 0.01 vs. control). Data are mean ± S.E.M., *n *= 20 in each group.

Figure 1A shows that mice receiving TBG‐Cre‐AAV8 had significantly lower serum IGF‐1 levels compared with control mice receiving TBG‐eGFP‐AAV8. Both groups had similar serum IGF‐1 levels prior to the administration of liver‐targeted viruses (data not shown). Consistent with the concept that circulating IGF‐1 contributes to the maintenance of IGF‐1 levels in the brain (Nishijima *et al*., 2010), we found that mice receiving TBG‐Cre‐AAV8 also had significantly lower tissue IGF‐1 levels in the cerebral cortex compared with control mice receiving TBG‐eGFP‐AAV8 (4.8 ± 1.7 and 11.4 ± 2.4 pg mg^−1^ of tissue, respectively; *P *= 0.03).

Changes in CBF in the whisker barrel cortex in response to contralateral whisker stimulation were significantly attenuated in IGF‐1‐deficient mice (Fig. 1B), indicating that IGF‐1 deficiency leads to neurovascular uncoupling, mimicking the aging phenotype (Toth *et al*., 2014a). IGF‐1 deficiency could reduce functional hyperemia by impairing neural activity evoked by whisker stimulation. To examine this possibility, we recorded spontaneous and evoked neural activity in control and IGF‐1‐deficient mice. We found that the amplitude and frequency distribution of the electrocorticogram and the amplitude of the somatosensory field potentials produced by the activation of the whisker pad do not differ between control and IGF‐1‐deficient mice (Fig. 1C–D). Therefore, IGF‐1 deficiency is unlikely to contribute to impaired functional hyperemia by modulating the neural activity evoked by whisker stimulation.

Our recent studies demonstrate that selective experimental disruption of neurovascular coupling responses is associated with significant impairment of cognitive function, recapitulating neurological symptoms and signs observed in brain aging (Tarantini, Ungvari and Toth, manuscript in preparation 2015). In this study, for the hippocampal‐dependent spatial memory test, the control mice entered the novel arm more often than the IGF‐1‐deficient mice following the intertrial interval (Fig. 1C). The control mice also spent significantly more time in the novel arm than the previously visited arms, whereas the IGF‐1‐deficient mice spent significantly less time in the novel arm (Fig. 1D), indicating that neurovascular uncoupling induced by IGF‐1 deficiency is also associated with impaired spatial working memory and novelty‐seeking behavior. Previous studies using the Morris water maze (Trejo *et al*., 2007) and the Barnes maze (Sonntag and Csiszar, unpublished data, 2012) also yielded similar results, showing that IGF‐1‐deficient mice exhibit impaired spatial working memory. Learning and/or memory deficits were also observed in GH/IGF‐1‐deficient Lewis dwarf rats (Nieves‐Martinez *et al*., 2010), spontaneously dwarf SD rats (Li *et al*., 2011), and in Ames dwarf mice in some (Derenne *et al*., 2011), but not all (Sharma *et al*., 2010), studies.

### IGF‐1 deficiency impairs cerebromicrovascular endothelial function: role in neurovascular uncoupling

**Figure 2:**
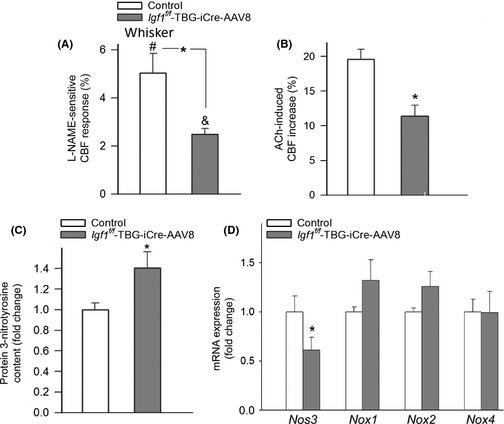
IGF‐1 deficiency impairs cerebromicrovascular endothelial function: role in neurovascular uncoupling. (A) L‐NAME‐sensitive, NO‐mediated portion of the CBF response (calculated based on the percentage decline in CBF in the presence of L‐NAME) measured above the barrel field of the primary somatosensory cortex in response to whisker stimulation in control and IGF‐1‐deficient (*Igf1* ^*f/f*^ + TBG‐Cre‐AAV8) mice (*n *= 6, * *P* < 0.05 vs. control; ^#^ *P* < 0.05 vs. control w/o drug; ^&^ *P* < 0.05 vs. *Igf1* ^*f/f*^ + TBG‐Cre‐AAV8 w/o drug). (B) CBF responses elicited by topical administration of acetylcholine to the barrel field of control and IGF‐1‐deficient mice (*n *= 6, *P < 0.05 vs. control). (C) Protein 3‐nitrotyrosine content, a biomarker of increased ONOO‐ formation, in cortical tissue of IGF‐1‐deficient and control mice (*n *= 5, *P < 0.05 vs. control). (D) qPCR data showing mRNA expression of the endothelial nitric oxide synthase (*Nos3*) and the NADPH oxidase subunits *Nox1*,* Nox2,* and *Nox4* in cortical samples of IGF‐1‐deficient and control mice. Data are mean ± S.E.M. (*n *= 5, *P < 0.05 vs. control).

We found that in control animals, the administration of the NO synthase inhibitor L‐NAME significantly decreased CBF responses in the barrel cortex elicited by contralateral whisker stimulation (Fig. 2A). In IGF‐1‐deficient animals, the effect of L‐NAME was blunted (Fig. 2A), suggesting that IGF‐1 deficiency impairs NO mediation, which contributes to neurovascular uncoupling. Topical application of the endothelium‐dependent vasodilator agent acetylcholine (ACh; 10^−5^ mol L^−1^) resulted in a significant increase in CBF in the barrel cortex of control mice (Fig. 2B). ACh‐induced CBF responses were significantly attenuated in IGF‐1‐deficient mice (Fig. 2B), supporting the concept that IGF‐1 deficiency impairs cerebromicrovascular endothelial function, mimicking the aging phenotype (Toth *et al*., 2014a). Previously, it has been found that in models of vascular aging in the periphery, IGF‐1 decreases vascular oxidative stress and improves endothelial function (Ungvari & Csiszar, 2012), whereas it does not significantly affect endothelium‐independent vasodilation elicited by NO donors (Bailey‐Downs *et al*., 2012). We found that in IGF‐1‐deficient mice, 3‐nitrotyrosine content in the cerebral cortex is significantly elevated (Fig. 2C) consistent with increased oxidative/nitrosative stress in the brain, which mimics the aging phenotype (Toth *et al*., 2014a). IGF‐1 deficiency was associated with decreased expression of *Nos3* (Fig. 2D). In IGF‐1‐deficient mice, the expression of *Nox1* and *Nox2* subunits of the NADPH oxidase tended to increase; however, the differences did not reach statistical significance (Fig. 2D).

### IGF‐1 deficiency impairs glutamate‐mediated CBF responses: role in neurovascular uncoupling

**Figure 3:**
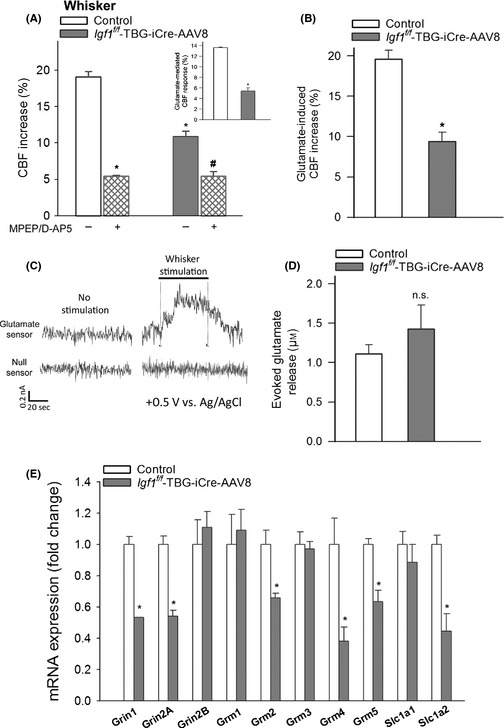
IGF‐1 deficiency impairs glutamate‐mediated CBF responses: role in neurovascular uncoupling. (A) Effects of treatment with antagonists of metabotropic glutamate receptors (MPEP, 5 × 10^−5^ mol L^−1^) and NMDA receptors (N‐methyl‐D‐aspartate, D‐APV, 5 × 10^−5^ mol L^−1^) on cerebral blood flow (CBF) responses measured above the barrel field of the primary somatosensory cortex in response to whisker stimulation in control and IGF‐1‐deficient mice (*Igf1* ^*f/f*^ + TBG‐Cre‐AAV8). The inlet shows the glutamate‐mediated part of the neurovascular response in each group (*n *= 6 in each group, *P < 0.05 vs. control; ^#^ *P* < 0.05 vs. *Igf1* ^*f/f*^ + TBG‐Cre‐AAV8). (B) CBF responses measured above the barrel field of the primary somatosensory cortex elicited by topical administration of L‐glutamate (500 μmol L^−1^) in control and IGF‐1‐deficient mice (*n *= 6 in each group, *P < 0.05 vs. control). Panel C Original recordings of changes in extracellular glutamate in response to whisker stimulation (5 Hz, 2 min) measured by amperometry using a glutamate biosensor inserted into the barrel cortex of mice (see Methods for details). ‘Null sensor’ indicates a biosensor constructed the same way as the glutamate sensors but without any enzymes for biosensing. Summary data are shown in Panel D. No significant differences (*P* = 0.4) were observed between cortical glutamate signals induced by whisker stimulation in control (*n *= 5) and IGF‐1‐deficient mice (*Igf1* ^*f/f*^ + TBG‐Cre‐AAV8, *n *= 7). (E) qPCR data showing mRNA expression of NMDA receptors (*Grin1*,* Grin2A*,* Grin2B*), metabotropic glutamate receptors (*Grm1*,* Grm2*,* Grm3*,* Grm4*,* Grm5*), and glutamate transporters (*Slc1a1*,* Slc1a2*) on astrocytes isolated from control and IGF‐1‐deficient animals (*n *= 5). *P < 0.05 vs. control. Data are mean ± S.E.M. for every panel of the figure.

Astrocytes were suggested to sense neuronal‐derived glutamate through metabotropic glutamate receptors (mGluR) and NMDA receptors, leading to increased production of vasodilator gliotransmitters that contribute to neurovascular coupling (Petzold & Murthy, 2011). In support of this concept, we found that in control mice, the metabotropic Glu receptor blocker MPEP and NMDA receptor blocker D‐APV significantly decreased CBF responses in the barrel cortex elicited by contralateral whisker stimulation (Fig. 3A). In IGF‐1‐deficient animals, the effect of MPEP plus D‐APV was significantly decreased (Fig. 3A), suggesting that IGF‐1 deficiency impairs glutamate‐mediated neurovascular coupling. Further support for this concept is provided by the findings that in IGF‐1‐deficient mice, glutamate‐induced CBF changes were also significantly impaired (Fig. 3B). IGF‐1 deficiency did not alter glutamate release induced by neuronal activation (Fig. 3C–D), whereas it dysregulated the astrocytic expression of metabotropic glutamate receptors (*Grm2*,* Grm4*,* Grm5)* and NMDA receptors (*Grin1* and *Grin2;* Fig. 3E).

### IGF‐1 deficiency impairs mediation of CBF responses by eicosanoid gliotransmitters: role in neurovascular uncoupling

**Figure 4:**
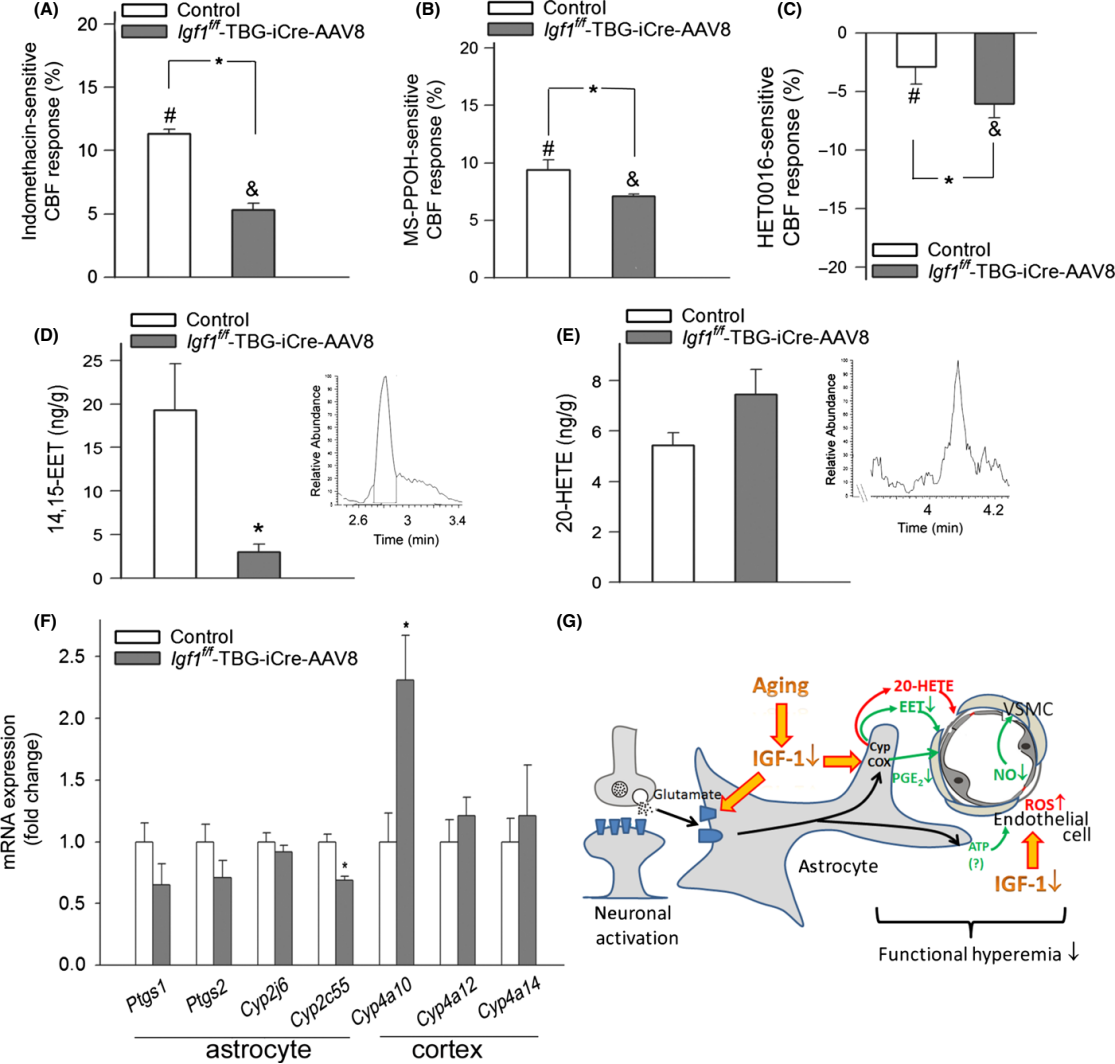
IGF‐1 deficiency impairs mediation of CBF responses by eicosanoid gliotransmitters: role in neurovascular uncoupling. (A) Indomethacin‐sensitive, prostaglandin‐mediated portion of the CBF response (calculated based on the percentage decline in CBF in the presence of INDO) measured above the barrel field of the primary somatosensory cortex in response to whisker stimulation in control and IGF‐1‐deficient (*Igf1* ^*f/f*^ + TBG‐Cre‐AAV8) mice (*n *= 6 in each group, *P < 0.05 vs. control; ^#^ *P* < 0.05 vs. control w/o INDO; ^&^ *P* < 0.05 vs. *Igf1* ^*f/f*^ + TBG‐Cre‐AAV8 w/o INDO). (B) MS‐PPOH‐sensitive, EET‐mediated portion of the CBF response (calculated based on the percentage decline in CBF in the presence of MS‐PPOH) measured above the barrel field of the primary somatosensory cortex in response to whisker stimulation in control and IGF‐1‐deficient (*Igf1* ^*f/f*^ + TBG‐Cre‐AAV8) mice (*n *= 6 in each group, *P < 0.05 vs. control; ^#^ *P* < 0.05 vs. control w/o MS‐PPOH; ^&^ *P* < 0.05 vs. *Igf1* ^*f/f*^ + TBG‐Cre‐AAV8 w/o MS‐PPOH). (C) HET0016‐sensitive, 20‐HETE‐mediated portion of the CBF response (calculated based on the percentage decline in CBF in the presence of the cytochrome P450 ω‐hydroxylase inhibitor HET0016) measured above the barrel field of the primary somatosensory cortex in response to whisker stimulation in control and IGF‐1‐deficient (*Igf1* ^*f/f*^ + TBG‐Cre‐AAV8) mice (*n *= 6 in each group, *P < 0.05 vs. control; ^#^ *P* < 0.05 vs. control w/o HET0016; ^&^ *P* < 0.05 vs. *Igf1* ^*f/f*^ + TBG‐Cre‐AAV8 w/o HET0016). (D–E) Production of 14,15 EET (D) and 20‐HETE (E) in glutamate‐activated brain slices from control and *Igf1* ^*f/f*^ + TBG‐Cre‐AAV8 mice as measured by liquid chromatography/mass spectrometry (LC/MS) (*n *= 6 in each group; *P < 0.05 vs. control; see Methods). (F) qPCR data showing mRNA expression of cyclooxygenase‐1 and cyclooxygenase‐2 (*Ptgs1*,* Ptgs2*) and EET‐producing epoxygenases (*Cyp2j6*,* Cyp2c55*) in isolated astrocytes, and 20‐HETE‐producing ω‐hydroxylases (*Cyp4a10*,* Cyp4a12*,* Cyp4a14*) in cortical samples of control and IGF‐1‐deficient mice (*n *= 5 in each group). *P < 0.05 vs. control. Data are mean ± S.E.M. (G) Proposed mechanisms by which age‐related IGF‐1 deficiency may impair neurovascular coupling responses (see Discussion). The model predicts that IGF‐1 deficiency both alters astrocytic production of eicosanoid gliotransmitters and impairs cerebromicrovascular endothelial function.

Upon activation by neuronal‐derived glutamate, astrocytes were shown to convert arachidonic acid by cyclooxygenases to vasodilator prostaglandins and by P450 epoxygenase to vasodilator EETs (Petzold & Murthy, 2011). Consistent with this concept, we found that in control animals, the administration of the cyclooxygenase inhibitor indomethacin (Fig. 4A) and P450 epoxygenase inhibitor MS‐PPOH (Fig. 4B) significantly decreased CBF responses in the barrel cortex elicited by contralateral whisker stimulation. In IGF‐1‐deficient animals, the effects of indomethacin (Fig. 4A) and MS‐PPOH (Fig. 4B) were significantly decreased, suggesting that IGF‐1 deficiency impairs the mediation of neurovascular coupling by prostaglandins and EETs. The astrocyte‐derived vasoconstrictor eicosanoid 20‐HETE can also negatively impact neurovascular coupling. Using the cytochrome P450 ω‐hydroxylase inhibitor HET0016, we found that IGF‐1 deficiency tended to increase the HET0016‐sensitive component of the CBF response, indicating that a 20‐HETE‐dependent constrictor response is present in IGF‐1‐deficient mice (Fig. 4C). LC/MS/MS measurements demonstrated that IGF‐1 deficiency resulted in a diminished cerebral production of the potent vasodilator 14,15‐EET (which is the most prevalent among the four EET regioisomers in the murine brain) in response to glutamate stimulation of brain slices (Fig. 4D). IGF‐1 deficiency also tended to increase the cerebral production of 20‐HETE (Fig. 4E). Among the investigated factors known to be involved in the synthesis of eicosanoid gliotransmitters, the expression of *Cyp2c55* decreased, whereas the expression of the 20‐HETE‐producing ω‐hydroxylase *Cyp4a10* increased in IGF‐1‐deficient mice (Fig. 4F).

## Discussion

Circulating IGF‐1 concentrations decrease significantly with age, due to decreases in GH levels, both in humans and in laboratory animals (Sonntag *et al*., 2005, 2013). There is substantial evidence that circulating IGF‐1 is an important vascular protective factor and that the age‐related decline in IGF‐1 levels contributes to vascular aging, promoting atherogenesis and development of cardiovascular disease and stroke (reviewed recently in Ungvari & Csiszar (2012) and Sonntag *et al*. (2013)). Here, we show for the first time that circulating IGF‐1 deficiency also leads to profound neurovascular dysregulation, characterized by impaired CBF responses induced by synaptic activity (Fig. 1), which mimics the cerebromicrovascular aging phenotype (Toth *et al*., 2014a). Impairment of a key homeostatic mechanism matching energy supply with the needs of active neuronal tissue is predicted to have deleterious effects on brain function. Indeed, there is strong evidence that in elderly patients, impaired neurovascular coupling (Zaletel *et al*., 2005; Topcuoglu *et al*., 2009; Stefanova *et al*., 2013) associates with decline in higher cortical functions including cognition. Importantly, neurovascular uncoupling in IGF‐1‐deficient mice also associates with impaired cognitive function (Fig. 1), mimicking the aging phenotype (Csiszar *et al*., 2013).

Although IGF‐1 deficiency may impact multiple aspects of neuronal function (Sonntag *et al*., 2013), our recent studies strongly suggest that a direct mechanistic link exists between neurovascular uncoupling and cognitive decline. Accordingly, recently we found that experimentally induced acute neurovascular uncoupling in mice, in the absence of alterations in synaptic function, leads to impaired performance in tests relevant for hippocampal‐ and cortical‐dependent tasks of learning and memory (Tarantini, Ungvari and Toth, manuscript in preparation 2015). In humans, IGF‐1 deficiency is associated with progressive cognitive dysfunction that can be reversed by increasing circulating IGF‐1 levels (reviewed in Sonntag *et al*. (2013)). Rodents have a similar decrease in circulating IGF‐1 levels with age and treatment of aged F344xBN rats with IGF‐1 was shown to improve cognitive function (reviewed in Sonntag *et al*. (2013)). Thus, further studies are warranted to determine whether in the aforementioned models treatment with IGF‐1 rescues neurovascular coupling and to establish a causal link between IGF‐1‐dependent changes in functional hyperemia and cognitive function. In addition to exerting protective effects on neurovascular coupling responses, IGF‐1 may also protect brain function by additional mechanisms, including exerting trophic effects on central glutamatergic synapses (Trejo *et al*., 2007) and/or preventing blood brain barrier disruption and neuroinflammation (Toth *et al*., 2014b).

The effects of IGF‐1 deficiency on the cellular mechanisms involved in neurovascular coupling are likely multifaceted. Microvascular endothelial cells are directly exposed to circulating IGF‐1 and are known to abundantly express IGF‐1 receptors (Ungvari & Csiszar, 2012). Importantly, there is growing experimental evidence that NO production by cerebromicrovascular endothelial cells has an important role in functional hyperemia (Girouard *et al*., 2007; Longden & Nelson, 2011; Stobart *et al*., 2013). This concept is supported by our observation that inhibition of NO synthesis significantly reduces neurovascular coupling in control animals (Fig. 2). It is significant that impaired endothelial NO production was shown to contribute to age‐related neurovascular uncoupling in mice (Park *et al*., 2007; Toth *et al*., 2014a). The findings that in IGF‐1‐deficient mice (Fig. 2), the L‐NAME‐sensitive, NO‐mediated portion of the neurovascular coupling response was decreased suggest that cerebromicrovascular endothelial dysfunction also contributes to neurovascular uncoupling in IGF‐1 deficiency (Park *et al*., 2007), mimicking the aging phenotype (Park *et al*., 2007; Toth *et al*., 2014a). The mechanisms by which IGF‐1 deficiency impairs cerebromicrovascular endothelial function likely involve an increased breakdown of NO by elevated levels of ROS. Several lines of evidence support this concept. First, IGF‐1‐deficient mice, similar to aged mice (Park *et al*., 2007; Toth *et al*., 2014a), exhibit increased production of ROS (Csiszar *et al*., 2008). Second, the treatment of primary endothelial cells with IGF‐1 significantly attenuates cellular ROS generation (Csiszar *et al*., 2008). Third, in the microcirculation, endothelium‐derived NO was shown to react with increased O_2_^.−^ forming ONOO^−^, thus decreasing the bioavailability of NO (Csiszar *et al*., 2002; Pacher *et al*., 2007). The brains of IGF‐1‐deficient mice, similar to aged mice (Toth *et al*., 2014a), exhibit an increased 3‐nitrotyrosine content (Fig. 2), a biomarker of increased ONOO^−^ formation, indicating that increased scavenging of vasodilator NO contributes to impaired endothelial mediation of cerebromicrovascular dilation in IGF‐1 deficiency. Previous studies suggest that increased activity/expression of NADPH oxidases and increased ROS production by mitochondrial sources contribute significantly to aging‐induced microvascular oxidative stress (Park *et al*., 2007; Ungvari & Csiszar, 2012; Toth *et al*., 2014a). Accordingly, the findings that treatment with a pharmacological inhibitor of cellular ROS production is able to improve functional hyperemia provide direct evidence for the role of increased oxidative stress in neurovascular uncoupling both in IGF‐1‐deficient mice and in aged mice (Toth *et al*., 2014a). Importantly, impaired endothelial function and increased oxidative stress are also manifest in the peripheral circulation in IGF‐1 deficiency (Gong *et al*., 2014) (reviewed in Ungvari & Csiszar (2012)). In addition to its vasodilator action, NO also confers multifaceted endothelial protective effects, including pro‐angiogenic, anti‐apoptotic, and anti‐inflammatory effects (Ungvari & Csiszar, 2012; Sonntag *et al*., 2013). Thus, it is likely that impaired endothelial function associated with IGF‐1 deficiency has complex phenotypic consequences in the cerebral microcirculation (e.g., alterations in microvascular density (Ungvari & Csiszar, 2012)), which should be elucidated by future studies.

In addition to the microvascular endothelial cells, the activation of astrocytic production of vasodilator arachidonic acid metabolites (EETs, prostaglandins) by neuronal‐derived glutamate also has an important role in neurovascular coupling (Petzold & Murthy, 2011). Here, we provide the first evidence that IGF‐1 deficiency alters the phenotype of astrocytes, impairing the astrocyte‐mediated portion of neurovascular coupling. We found that IGF‐1 deficiency impairs glutamate‐mediated gliovascular coupling responses (Fig. 3A), but it does not affect glutamate release from neurons (Fig. 3C), suggesting a primary astrocytic deficit. Our findings indicate that the likely mechanisms by which IGF‐1 deficiency impairs glutamate‐mediated gliovascular coupling responses include decreased expression of astrocytic glutamate receptors (Fig. 3D) and dysregulation of astrocytic synthesis of eicosanoid gliotransmitters, likely due to altered expression of cytochrome P‐450 enzymes in the astrocytes (Fig. 4). On the basis of the aforementioned findings, one would predict that restoration of circulating IGF‐1 levels in aging would positively impact astrocyte function, improving gliovascular coupling. This hypothesis needs to be experimentally tested in future studies. It should be noted that NO *per se* is likely to regulate the metabolism of arachidonic acid by cytochrome P‐450 enzymes in the vasculature, inhibiting the production of vasoconstrictor 20‐HETE. Thus, future studies should also determine whether decreased bioavailability of NO contributes to increased production of 20‐HETE in the brains of IGF‐1‐deficient mice.

In conclusion, our results add to the growing evidence that IGF‐1 exerts an important cerebromicrovascular protective effect (Toth *et al*., 2014b), which likely supports multiple aspects of brain health. The findings that isolated circulating IGF1 deficiency results in functional and phenotypic alterations in endothelial cells and astrocytes and leads to neurovascular uncoupling have important clinical relevance for cognitive impairment associated with both aging and genetic IGF‐1 deficiency (e.g., Laron dwarfism). Our findings, taken together with the results of earlier studies (reviewed in Sonntag *et al*. (2013)), point to potential benefits of interventions preventing age‐related IGF‐1 deficiency and promoting microvascular health for the prevention of cognitive decline in the elderly.

## Experimental procedures

All procedures were approved by and followed the guidelines of the Institutional Animal Care and Use Committee of OUHSC in accordance with the ARRIVE guidelines.

### Postdevelopmental liver‐specific knockdown of Igf1 in mice

Male mice homozygous for a floxed exon 4 of the *Igf1* gene (*Igf1* ^*f/f*^) in a C57BL/6 background were used (Toth *et al*., 2014b). These mice have the entirety of exon 4 of the *Igf1* gene flanked by loxP sites, which allows for genomic excision of this exon when exposed to Cre recombinase. Transcripts of the altered *Igf1* gene yield a protein upon translation that fails to bind the IGF receptor. Animals were housed in the Rodent Barrier Facility at OUHSC, on a 12‐h light/12‐h dark cycle, and given access to standard rodent chow (Purina Mills, Richmond, IN, USA) and water *ad libitum*. To target hepatocytes, adeno‐associated viruses (AAVs) were purchased from the University of Pennsylvania Vector Core (Philadelphia, PA, USA). At 2 months of age, approximately 1.3 × 10^10^ viral particles (as assayed by genome content at the University of Pennsylvania) of AAV8.TBG.PI.Cre.rBG or AAV8.TBG.PI.eGFP.WPRE.bGH were administered to *Igf1* ^*f/f*^ mice to knockdown IGF‐1 or as a control, respectively, as described (Toth *et al*., 2014b). While AAV8 is effective at transducing multiple tissues after i.v. delivery, including liver, the thyroxine‐binding globulin (TBG) promoter restricts the expression solely to hepatocytes, as described (Toth *et al*., 2014b). Experiments were conducted 3 months postknockdown of *Igf1*.

### Measurement of circulating and tissue IGF‐1 levels

Venous blood was collected from the submandibular veins of animals from both groups (Medipoint, Mineola, NY, USA). Whole blood was centrifuged at 2500 *g* for 20 min at 4°C to collect serum, which was then stored at −80°C. IGF‐1 levels in sera and cortical tissue samples were measured by ELISA (R&D Systems, Minneapolis, MN, USA) according to the manufacturer's protocol and are reported in ng mL^−1^ and pg mg^−1^ tissue, respectively.

### Spatial memory testing of mice in Y‐maze

Three months after IGF‐1 knockdown, animals were tested for spatial working memory in the Y‐maze as described (Csiszar *et al*., 2013). In brief, a Y‐maze apparatus, made up of three enclosed transparent Plexiglas arms (40 cm length × 9 cm width × 16 cm height) with extra‐maze visual cues around the maze, was used to assess hippocampal‐dependent spatial recognition memory. The test consisted of two trials separated by an intertrial interval (4 h). All mice were transported to the behavioral testing room in their home cages at least 1 h before testing. In the first training (acquisition) trial, mice were placed in the maze facing the end of a pseudorandomly chosen start arm and allowed to explore the maze for 5 min with one of the arms closed (novel arm). Mice were returned to their home cage until the second (retrieval) trial, during which they could explore freely all three arms of the maze. The time spent in each arm and number of entries were measured and analyzed from video recordings (Ethovision, Noldus Information Technology Inc., Leesburg, VA, USA). Mice were required to enter an arm with all four paws in order for it to be counted as an entry. The results are expressed as number of entries (entry) and time spent (dwell) in the novel arm as % of all entries and time spent in novel and other arm (Sarnyai *et al*., 2000). Entering the novel arm more frequently and for longer periods of time indicates intact memory and novelty‐seeking behavior because of the innate tendency of mice to explore. The maze was cleaned with 70% ethanol between the trials.

### Surgical procedures

Mice in each group were anesthetized with α‐chloralose (50 mg kg^−1^, i.p.) and urethane (750 mg kg^−1^, i.p.), endotracheally intubated, and ventilated (MousVent G500; Kent Scientific Co, Torrington, CT, USA). Rectal temperature was maintained at 37°C using a thermostatic heating pad (Kent Scientific Co). End‐tidal CO_2_ (including dead space) was maintained between 3.2% and 3.7% to keep blood gas values within the physiological range (PaCO_2_: 36.18 ± 1.8 mmHg, PaO2: 109.8 ±3.1 mmHg). Mice were immobilized, placed on a stereotaxic frame (Leica Microsystems Inc, Buffalo Grove, IL, USA), the scalp and periosteum were pulled aside and the skull was removed over the barrel cortex, and the dura was gently removed. The cranial window was superfused with artificial cerebrospinal fluid (ACSF, composition: NaCl 119 mm, NaHCO_3_ 26.2 mm, KCl 2.5 mm, NaH_2_PO_4_ 1 mm, MgCl_2_ 1.3 mm, glucose 10 mm, CaCl_2_ 2.5 mm, pH = 7.3, 37°C). The right femoral artery was cannulated for arterial blood pressure measurement (Living Systems Instrumentations, Burlington, VT, USA). The blood pressure was within the physiological range throughout the experiments (90–110 mmHg).

### Cerebral blood flow responses to whisker stimulation and pharmacological studies

To assess neurovascular coupling, a laser Doppler probe (Transonic Systems Inc., Ithaca, NY, USA) was placed above the barrel cortex (1–1.5 mm posterior and 3–3.5 mm lateral to bregma), and the contralateral whiskers were stimulated for 1 min at 5 Hz from side to side. Changes in CBF (*n *= 7–8 mic in each group) were assessed in three trials (5‐ to 10‐min intervals). CBF responses to whisker stimulation were repeated in the presence of the following inhibitors administered topically onto the brain surface of separate groups of animals: HET0016 (inhibitor of 20‐hydroxyeicosatrienoic acid (20‐HETE) production, 10^−6 ^mol L^−1^ for 30 min; Cayman Chemicals, Ann Arbor, MI, USA) (Liu *et al*., 2008), MS‐PPOH (inhibitor of EET production, 20 × 10^−6 ^mol L^−1^ for 30 min; Cayman Chemicals) (Shi *et al*., 2008), L‐NAME (N^ω^‐Nitro‐L‐arginine methyl ester, inhibitor of nitric oxide synthase, 10^−4 ^mol L^−1^ for 20 min; Sigma‐Aldrich, St. Louis, MO, U.S.A.), apocynin (inhibitor of NADPH oxidases, 3 × 10^−4 ^mol L^−1^ for 30 min; Cayman Chemicals), fluoroacetate sodium (inhibitor of the tricarboxylic acid cycle predominantly in glial cells, 10^−4^ mol L^−1^ min; Sigma‐Aldrich, St. Louis, MO, U.S.A.) (Fonnum *et al*., 1997; Lecrux *et al*., 2012), indomethacin (cyclooxygenase inhibitor, 5 × 10^−4^ mol L^−1^; Sigma‐Aldrich, St. Louis, MO, U.S.A.) (Kitaura *et al*., 2007), MPEP (6‐Methyl‐2‐(phenylethynyl)pyridine hydrochloride, group I metabotropic glutamate receptors (mGluR) subtype 5 antagonist, 5 × 10^−5^ mol L^−1^) (Zonta *et al*., 2003), and the NMDA (N‐methyl‐D‐aspartate) receptor antagonist D‐APV (D‐2‐Amino‐5‐Phosphonovaleric acid, 5 × 10^−5^ mol L^−1^; Cayman Chemicals) (Stobart *et al*., 2013). In a separate series of experiments (*n *= 8 in each group), CBF responses to topical administration of L‐glutamate (500 μmol L^−1^) (Hall *et al*., 2014) were determined in the absence and presence of MPEP (5 × 10^−5^ mol L^−1^) and D‐APV (5 × 10^−5^ mol L^−1^) (Stobart *et al*., 2013). CBF responses to acetylcholine (ACh; 10^−5^ mol L^−1^) were also obtained to assess maximal endothelial NO‐mediated responses. Changes in CBF are expressed as percent (%) changes from baseline.

### Spontaneous neuronal activity and evoked field potentials (SEP) in the primary somatosensory cortex

The animals were surgically prepared and ventilated as described above, and a glass‐insulated tungsten microelectrode (impedance, 2–3 MΩ; Kation Scientific, LLC, Minneapolis, MN, USA) was inserted into the left barrel cortex (1–1.5 mm posterior and 3–3.5 mm lateral to bregma) through the ACSF‐perfused open cranial window for recording local field potentials. An Ag/AgCl electrode inserted in the neck muscles served as reference. After basal activity was recorded, the right whisker pad was stimulated by a bipolar stimulating electrode placed to the ramus infraorbitalis of the trigeminal nerve and into the masticatory muscles. The stimulation protocol used to investigate neurovascular coupling and somatosensory evoked field potentials consisted of 10 stimulation presentation trials with an intertrial interval of 70 s, each delivering a 15‐s train of electrical pulses (2 Hz, 0.2 mA, intensity, and 0.3 ms pulse width) after a 10‐s prestimulation baseline period. The signal was amplified with an AC/DC differential amplifier (high pass at 1 Hz, low pass at 1 kHz) (Model 3000; A‐M Systems, Inc. Carlsborg, WA, USA) and digitalized by the PowerLab/Labchart data acquisition system (ADInstruments, Colorado Springs, CO, USA) with a sampling rate of 40 kHz. Basal activity was analyzed as distribution of wave amplitude as a function of frequency (Park *et al*., 2008), and the negative amplitude in the somatosensory evoked field potential response was considered as the excitatory postsynaptic potential (fEPSP) (Lind *et al*., 2013). Analyses were performed on the average of 10 stimulation trials.

### Cerebral glutamate release to whisker stimulation

In a separate cohort of animals, we assessed changes in extracellular glutamate signal in response to whisker stimulation. The cranial window was superfused continuously with ACSF. The glutamate sensor is a platinum electrode encapsulated in a biolayer containing glutamate oxidase and protected against the interference with ascorbate, urate, dopamine, and 5‐hydroxytriptamine. Glutamate is oxidized into hydrogen peroxide which is sensed by the electrode. In the morning of the experiments, the working electrode (Sarissa GLU Biosensor, 25 μm tip; Sarissa Biomedical, Coventry, UK) was calibrated according to the manufacturer's guidelines *in vitro*, and then, it was inserted 1.5 mm caudal and 3 mm lateral from bregma into the cerebral tissue about 500 μm deep. Glutamate null sensors (lack of any enzymes for biosensing) were us ed as controls. The reference electrode (Ag/AgCl) was inserted into the cerebral tissue elsewhere, and the auxiliary electrode (Ag/AgCl) was placed between the scalp and the skull. The potential was set at 0.5 V vs. Ag/AgCl. A 3‐electrode potentiostat (Quadstat) with an eDAQ data acquisition system (eDAQ Pty Ltd., Colorado Springs, CO, USA) was used for constant potential amperometry. Following the insertion of the electrodes, we waited about 2‐3 min until a stable baseline developed. Then, we stimulated the right whiskers for 1 min at 5 Hz, in three consecutive trials divided by 5‐ to 10‐min intervals. The response was recorded in nA and converted to μM of glutamate using the calibration curve. Basal glutamate measurements preceding the evoked glutamate signal were included only, and amplitudes were calculated by obtaining the maximum increase from baseline (Onifer *et al*., 2012).

### Astrocyte isolation

Astrocytes were immunopurified from the cortex of the experimental animals by targeting the extracellular epitope of *Glast*, a glutamate transporter specifically found on astrocytes. Cortices were isolated in ice‐cold HBSS, enzymatically digested in L15 media with 0.05% trypsin for 20 min at 37°C, titurated, and filtered through 0.7‐μm mesh cell strainer. The remaining cells were pelleted, resuspended in 100 μls of HBSS with 0.5% BSA, and incubated with anti‐Glast‐PE (1:10; Miltenyi Biotech, Bergisch Gladbach, Germany) for 10 min at 4°C. Anti‐PE microbeads (Miltenyi Biotech) were then added and incubated for an additional 10 min at 4°C. Cells were pelleted and washed three times with PBS with 0.5% BSA before magnetic bead column purification (Miltenyi Biotech).

### Quantitative real‐time RT–PCR

A quantitative real‐time RT–PCR technique was used to analyze mRNA expression of nitric oxide synthase, NADPH oxidases, NMDA receptors, metabotropic glutamate receptors, cyclooxygenases, epoxygenases, and ω‐hydroxylases in cortical samples and isolated astrocytes from each experimental group using a Strategen MX3000 platform, as previously reported (Toth *et al*., 2013b). In brief, total RNA was isolated with a Mini RNA Isolation Kit (Zymo Research, Orange, CA, USA) and was reverse‐transcribed using Superscript III RT (Invitrogen, Burlington, ON, Canada) (Toth *et al*., 2013a,b). Amplification efficiencies were determined using a dilution series of a standard vascular sample. Quantification was performed using the efficiency‐corrected ΔΔCq method. The relative quantities of the reference genes *Hprt, Ywhaz, B2 m,* and *Actb* were determined, and a normalization factor was calculated based on the geometric mean for internal normalization. Fidelity of the PCR was determined by melting temperature analysis and visualization of the product on a 2% agarose gel.

### Measurement of the production of arachidonic acid metabolites in brain slices

To determine how IGF‐1 deficiency affects the synthesis of eicosanoid gliotransmitters, horizontal hippocampal slices of 400 μm thickness from mice in each cohort were prepared in ice‐cold solution containing (in mmol L^−1^) sucrose 110, NaCl 60, KCl 3, NaH_2_PO_4_ 1.25, NaHCO_3_ 28, sodium ascorbate acid 0.6, glucose 5, MgCl_2_ 7, and CaCl_2_ 0.5 using a HM650V vibrating microtome (Thermo Scientific, Burlington, ON, Canada). Slices were then transferred to a custom‐made chamber which contained oxygenated artificial cerebrospinal fluid (aCSF) of the following composition (in mm): NaCl 126, KCl 2.5, NaH_2_PO_4_ 1.25, MgCl_2_ 2, CaCl_2_ 2, NaHCO_3_ 26, glucose 10, pyruvic acid 2, and ascorbic acid 0.4. To stimulate astrocytes, glutamate (3 × 10^−4^ mol L^−1^) was added to the chamber. The samples were weighed and snap‐frozen for further analysis.

The samples were homogenized in ice‐cold phosphate buffer (pH 6.8). Thousand units of *E. coli* β‐glucuronidase was added to the tissue extract to release 20‐HETE from conjugation with glucuronide. After incubation at 37°C for 2 h, the pH in the solution was adjusted to 3 by the addition of acetic acid. [^2^H_4_]‐20‐HETE (10 ng) was added, and the sample was extracted with acidified CHCl_3_/CH_3_OH (2:1) and purified by silica solid‐phase extraction. Different samples were placed into ice‐cold 0.15M KCl. After homogenization, synthetic [^2^H_11_]‐labeled 14,15‐DHET (5 ng) was added as internal standard. The EETs and DHETs were extracted from the tissue homogenates with acidified CHCl_3_/CH_3_OH (2:1) and purified by silica solid‐phase extraction, separating EETs and DHETs.

Quantification was performed by LC/MS/MS using Acquity BEH C18 columns (1.0 × 100 mm; 1.7 μm) connected to a TSQ‐Quantum Vantage triple quadrupole spectrometer (ThermoScientific) with a linear solvent gradient that went from 70% 15 mm aqueous ammonium acetate (pH 8.5), 30% acetonitrile to 40% 15 mm aqueous ammonium acetate (pH 8.5), 60% acetonitrile in 6 min and at a flow of 0.18 mL/min. For 20‐HETE analysis, we utilized collision‐induced fragmentation of 20‐HETE at m/z 319 and the [^2^H_4_]‐20‐HETE internal standard at m/z 325. The ratio of the area of the 20‐HETE peak compared to the area of the corresponding deuterated 20‐HETE was used for quantification. For EET analysis, the EETs were converted to the corresponding DHETs by treatment with acetic acid overnight. Then, we utilized collision‐induced fragmentation of the DHETs at m/z 337 and the [^2^H_11_]–DHET internal standards at m/z 448. Diagnostic selective product ion analysis was performed at m/z 206 for 14,15‐DHET. These same product ions were also used for the deuterated internal standards. Quantifications were performed using the ratio of the area of the DHET peaks compared to the area of the corresponding deuterated DHET peaks (Capdevila *et al*., 1991; Nakagawa *et al*., 2006).

### Determination of cerebral oxidative stress

To characterize the effect of IGF‐1 deficiency on cerebral oxidative stress, the marker for peroxynitrate action, 3‐nitrotyrosine (3‐NT), was assessed in homogenates of cortical samples using OxiSelect Protein Nitrotyrosine ELISA Kits (Cell Biolabs, San Diego, CA, U.S.A.) following the manufacturer's guidelines, as previously described (Toth *et al*., 2013b).

### Statistical analysis

Statistical analysis was carried out by unpaired t‐test or two‐way ANOVA for repeated measures followed by Bonferroni multiple comparison test, as appropriate, using Prism 5.0 for Windows (Graphpad Software, La Jolla, CA, USA). A *P* value <0.05 was considered statistically significant. Data are expressed as mean ± S.E.M.

## Funding

No funding information provided.

## Conflict of interest

The authors declare no competing financial interests.

## Author contribution

PT, ST, NMA, MNVA, GLM, WES, AC, and ZU designed research and revised the manuscript; PT, ST, NMA, MNVA, GLM, AM, AC, and ZU performed experiments; PT, ST, AC, GLM, NMA, and ZU analyzed data; and PT, AC, and ZU wrote the manuscript.
